# Demographic turnover can be a leading driver of hierarchy dynamics, and social inheritance modifies its effects

**DOI:** 10.1098/rstb.2022.0308

**Published:** 2023-08-14

**Authors:** Eli D. Strauss

**Affiliations:** ^1^ Centre for the Advanced Study of Collective Behaviour, University of Konstanz, Konstanz, Baden-Württemberg, 78464, Germany; ^2^ Ecology of Animal Societies Department, Max Planck Institute of Animal Behavior, Radolfzell, Baden-Württemberg, 78315, Germany; ^3^ Collective Behavior Department, Max Planck Institute of Animal Behavior, Radolfzell, Baden-Württemberg, 78315, Germany; ^4^ Integrative Biology Department, Michigan State University, East Lansing, Michigan, 48824, USA

**Keywords:** inequality, social ageing, social inheritance, reproductive skew, demography, social mobility

## Abstract

Individuals and societies are linked through a feedback loop of mutual influence. Demographic turnover shapes group composition and structure by adding and removing individuals, and social inheritance shapes social structure through the transmission of social traits from parents to offspring. Here I examine how these drivers of social structure feedback to influence individual outcomes. I explore these society-to-individual effects in systems with social inheritance of hierarchy position, as occur in many primates and spotted hyenas. Applying Markov chain models to empirical and simulated data reveals how demography and social inheritance interact to strongly shape individual hierarchy positions. In hyena societies, demographic processes—not status seeking—account for the majority of hierarchy dynamics and cause an on-average lifetime decline in social hierarchy position. Simulated societies clarify how social inheritance alters demographic effects—demographic processes cause hierarchy position to regress to the mean, but the addition of social inheritance modifies this pattern. Notably, the combination of social inheritance and rank-related reproductive success causes individuals to decline in rank over their lifespans, as seen in the hyena data. Further analyses explore how ‘queens’ escape this pattern of decline, and how variation in social inheritance generates variability in reproductive inequality.

This article is part of the theme issue ‘Evolutionary ecology of inequality’.

## 1. Introduction

Individuals and societies are linked in a feedback loop of mutual influence, where individuals shape the structure of their society through behaviour, and their behaviours and traits are in turn influenced by their societies [1]. For instance, through reproduction, mortality and dispersal, individuals modify the structure and composition of their groups [2]. Although much work has focused on the ways individuals shape social structure [3,4], the influences of social structure on individuals and their traits are less well understood. Society-to-individual effects occur when individual outcomes are impacted by the structures at the group level rather than by interactions with any specific group-mates in particular. For instance, the recent COVID-19 pandemic has highlighted how social network structure beyond our immediate contacts can shape our pathogen exposure, a phenomenon that is also characteristic of animal societies [5]. Outside of pathogen exposure and cultural transmission, there are few areas of behavioural biology where there exists an integrative understanding of how processes that structure societies feed back to shape individuals [1].

One remarkably widespread feature of societies is inequality, where group members benefit differentially from social living, for instance through unequal access to social partners or resources [6]. Individual position in gradients of social benefit are hypothesized to exert a strong influence on health and fitness of individuals [7]. In non-human animals, these gradients are often understood through the lens of dominance hierarchies, or networks of dominance relationships that structure how individuals resolve conflicts of interest within societies [8–10]. Some structural aspects of social hierarchies are remarkably consistent across species [11], whereas others vary considerably among species; examples include the determinants of high status [12,13], the use of social information in competition with group-mates [14,15], the steepness of the dominance hierarchy [16,17], and the ways in which hierarchy position influences individual health and reproduction [18].

A process determining position within these gradients is social inheritance, where social traits are passed from parents to offspring through non-genetic transmission [19]. One of the most striking types of social inheritance among non-human animals occurs in spotted hyenas and cercopithecine primates; in these species, position in the dominance hierarchy, and in some cases also in the broader social network ([20,21], but see [22]), is passed from mother to offspring [23–26]. The transmission of social traits from parents to offspring represents a behavioural mechanism by which individuals shape the structure of their societies [2,19,27], but more work is needed to trace how social inheritance feeds back to impact individuals through society-to-individual effects [1]. Any such effects need to be incorporated into a broader framework of demographic processes, as social inheritance occurs during the addition of new individuals.

Social inheritance and demographic turnover might feed back to impact individuals by driving changes in the social hierarchy. Social hierarchies are dynamic, and studying their dynamics provides a strong leverage point for understanding their impact on individuals [28]. For instance, recent work on the dynamics of social hierarchies have driven new insight into neural and cognitive mechanisms of social hierarchies [29], physiological consequences of current and past position in the social hierarchy [30,31], the emergence of new hierarchies [32–34] and the effects of social hierarchies on other social domains [35].

Two types of processes produce changes in individual position in the social hierarchy. Active dynamics refer to changes where individuals overtake others within the social gradient through status seeking behaviour [36]. Individuals can achieve these changes on their own [32,37], or they may form coalitions with other group-mates to challenge and overtake other members of their group [38–41]. By contrast, passive dynamics occur via demographic processes, such as the addition and subtraction of members from the group [42]; for instance, when Queen Elizabeth II passed away, Charles III became King of the United Kingdom. The key distinction between these two types of dynamics is that active dynamics occur because of the reversal of a previous order in the social hierarchy, whereas passive processes occur in the absence of changes to any dyadic order relationships within the group. Together, these two types of dynamics account for all individual-level changes in position in the social hierarchy, but passive dynamics have received less attention than active dynamics in studies of social hierarchies [36].

Here I use Markov chain models to characterize the social hierarchy dynamics in spotted hyenas (*Crocuta crocuta*) and examine how demographic turnover and social inheritance interact to influence hierarchy position. First, using empirical data from four groups of spotted hyenas, I disentangle the contributions of active and passive dynamics to the total dynamics that individuals experience. I use transition matrices inferred from Markov chain models of observed dynamics to generate expected lifetime dominance trajectories based on starting position. This Markov chain approach is inspired by studies of social mobility in humans, and has the advantage of being able to infer expected lifetime dominance trajectories from input data representing incomplete lifetimes. Second, to understand the role of social inheritance in driving the results observed in the empirical hyena data, I simulate societies with and without social inheritance, and apply the same Markov chain analysis to hierarchy dynamics in these societies. Finally, I simulate societies with different variants of social inheritance of hierarchy position to explore how variation in social inheritance produces variation in expected inequality in reproductive output.

## 2. Methods

### (a) Empirical study system

Spotted hyenas have convergently evolved the nepotistic hierarchical societies found in many cercopithecine primates [23]. Nepotistic societies are characterized by female philopatry, behavioural inheritance of position in the hierarchy, and kin structured social bonds [43–49]. Social inheritance of hierarchy position follows Kawamura's two rules—matrilineal cohesion, where descendants of the same female are grouped together in the hierarchy, and youngest ascendency, where younger offspring occupy higher positions than their older siblings [24,25]. However, there exists both within- and among-species variation in the extent to which cercopithecine primates follow these two rules [50–53] and the extent to which hierarchy position is associated with reproductive or other benefits [44,54,55]. Spotted hyenas predictably follow these rules, making them an ideal study system for understanding the structural properties of nepotistic societies [56].

Data were collected by the Mara Hyena Project from four groups of wild spotted hyenas in the Masai Mara National Reserve between the years of 1988 and 2018. Near-daily observations were conducted on study animals around dawn and dusk, when demographic and behavioural data were collected using scan and all-occurrence sampling [57] (see [58] for more details). Of importance for this study, agonistic interactions were recorded whenever they occurred in the presence of observers. These interactions are defined by the offering of a subordination behaviour, either spontaneously or in response to a threat. The receiver of the subordination signal was considered the winner of the interaction, and the loser of the interaction was defined as the individual offering the signal.

A longitudinal social hierarchy was inferred based on the winners and losers of these agonistic interactions. Positions in the social hierarchy were calculated yearly for all individuals who were at least 13 months of age on 1 January, because prior results indicate this is around the age at which juveniles begin winning and losing interactions in a manner similar to adults [56]. Hierarchies were updated yearly based on demographic changes and the outcomes of observed interactions in that year (see [36] for details). The hierarchy included natal females and males; male dispersal is the norm in this system, and after dispersal, males join new clans at the very bottom of the social hierarchy, ranking below all natal individuals [59]. Active dynamics were calculated as changes resulting from reversal of prior orderings, and passive dynamics were calculated by subtracting active dynamics from total dynamics [36]. Ranks and dynamics were calculated separately for each of the four groups and were standardized by group size to range from 0 (lowest) to 1 (highest). Standardized rank best captures hierarchical regulation of competition that is independent of group size [60]. Standardizing rank is a common approach in studies of spotted hyenas (e.g. [61,62]), where competition is decoupled from overall group size because of fission-fusion dynamics in which group-mates associate in smaller subgroups that change composition repeatedly throughout the day [63]. Using a paired *t*-test, I compared the relative contributions of active and passive processes to overall dynamics by taking the absolute value of the net rank displacement in hierarchy position for 350 individuals with complete tenures in the group.

### (b) Markov chains of hierarchy dynamics in empirical data

To understand drivers of hierarchy dynamics, I used Markov chains to model changes in hierarchy position according to an individual's starting position. Specifically, I fit discrete-time Markov chains using each decile in the social hierarchy as a state variable (10th decile = highest ranking 10%, 1st decile = lowest ranking 10%). This approach is inspired by classical studies of social mobility in humans [64,65], and is similar to an approach that has been used to model social hierarchy dynamics in Tibetan macaques (*Macaca thibetana*) [66]. Markov chain models are simple and elegant stochastic models that estimate the probability of transitioning to any of a set of potential states given only a current state [67]. This approach assumes that transition probabilities are not influenced by prior history or any persistent differences among individuals. Although this assumption is probably violated in this and other systems in which these approaches are applied, it is useful here for examining hierarchy dynamics in the absence of any individual differences in behaviour or any effects of historical changes. I chose this approach over alternative approaches like linear models of hierarchy dynamics because Markov chains can generate state sequences of arbitrary length from finite-length input data. In the context of this study, this approach allows me to use hierarchy dynamics data from incomplete or short lifespans to estimate transition probabilities between different parts of the hierarchy, then use those transition probabilities to generate expected trajectories of any defined length. Markov chains were fitted using the *markovchain* R package [68].

I first fitted Markov chains using the all the observed sequences of hierarchy position decile states from all four groups of spotted hyenas to quantify total hierarchy dynamics. This model estimated transition probabilities among the different states, and I used these transition probabilities to generate two summaries of hierarchy dynamics. First, I used the transition probabilities to generate sequences of deciles for individuals starting in different states—these sequences represent expected lifetime trajectories in hierarchy position. For each starting decile, I generated 10 000 sequences of state transitions, and summarized the mean and standard deviation of expected hierarchy position over time. Each sequence was 10 years long (i.e. 10 transitions), corresponding to a slightly above average female reproductive lifespan [55,69]. Second, I used the transition probability matrices to identify steady state distributions of hierarchy positions by taking incrementally higher powers of the transition matrix (i.e. multiplying the matrix by itself) until all entries change by less than 0.001 [66,67]. These steady states reflect the equilibrium distribution towards which the Markov chain approaches in the long run, and represent the probability of an individual occupying each state at a given time step if they lived an infinitely long time. These steady state distributions are a mathematical property of the Markov process, and rank distributions in a group will never actually reach these steady states because individuals have finite lifespans and because of demographic turnover in the group. Nevertheless, they are a useful summary of the hierarchy positions that individuals are drawn towards based upon the transition probabilities estimated by the Markov chain.

To understand the contributions of active and passive processes to total hierarchy dynamics, I repeated the Markov chain model and subsequent analysis with two different sets of input data that reflect the active and passive components of the hierarchy dynamics observed in the hyena societies. I decomposed each observed change in hierarchy position into its active and passive components (see [36] for details), then added all the active dynamics each individual experienced to their starting hierarchy position. This produced hierarchy position sequences that each individual would have underwent had they experienced only their active dynamics and none of their passive dynamics. I repeated the same process for passive dynamics, then fitted Markov chain models to these new datasets of hierarchy position sequences arising from the two different types of dynamics. As with the Markov chain model of total dynamics, I used these models of active and passive dynamics to generate expected lifetime trajectories and steady state probabilities summarizing the effects that these dynamics have on individual hierarchy position.

### (c) Simulated societies exploring how social inheritance shapes demographic effects

I next simulated societies to interrogate the role of social inheritance in driving the patterns observed in the empirical data. In particular, the prior analyses indicated that demographic processes produce strong effects on individual hierarchy position through passive dynamics. To understand how social inheritance influences these demographic effects, I simulated societies with and without social inheritance. I also varied the extent to which hierarchy position influences reproductive success. In total, I simulated 100 replicates of four types of societies accounting for all combinations of the presence and absence of social inheritance of hierarchy position and rank effects on reproduction. Unlike the empirical data that included both active and passive dynamics, here I simulated no active dynamics, such that all changes in these simulations were owing to passive processes.

All simulations shared the same basic architecture. Social groups were initialized with 30 individuals, and at each update, one individual was chosen to reproduce and one was chosen to suffer mortality. For simplicity, sex was ignored in these simulations, reproduction was asexual, and the probability of mortality was uniform across positions in the social hierarchy. Because in nepotistic mammalian societies adult males typically disperse to join new groups, these simulations most closely model the experiences of females and males prior to dispersal.

The details of reproduction varied among simulations according to whether there was social inheritance of hierarchy position and whether hierarchy position influenced reproductive success. To simulate social inheritance of rank, new individuals were assigned a position in the hierarchy according to the rules of maternal rank inheritance with youngest ascendancy (i.e. directly below the mother and above any older siblings [25]). In the absence of social inheritance, new individuals were added to the hierarchy at random. The effect of social hierarchy position on fitness was parameterized based on data on the annual rate of offspring production from the long-term hyena data (electronic supplementary material). This is a conservative estimate of the effects of hierarchy position on reproductive success, as it does not include the effects of hierarchy position on age at first reproduction [55].

I then repeated the Markov chain analysis from the empirical hyena data. I fitted Markov chains to the hierarchy dynamics observed in the simulated societies, then used the estimated transition matrices to generate lifetime dominance trajectories for individuals starting in different parts of the hierarchy. For these chains, I generated 10 000 trajectories that were 120 updates long (i.e. 120 recruitment events), which approximates the number of recruitment events occurring in a group of 30 individuals over a 10 year reproductive lifespan (electronic supplemental material). Finally, to examine potential ‘queenship’ effects of occupying the highest-ranking position in society, I reran these analyses treating queen status as an additional 11th state in the Markov chain.

### (d) Social inheritance variants and reproductive inequality

The prior analyses investigated social inheritance via maternal rank inheritance, but this is not the only form of social inheritance of hierarchy position. To understand how different variants of social inheritance might impact the observed patterns, I simulated societies where rank influences reproductive success but with two alternative types of social inheritance of hierarchy position. In the first, individuals inherit their rank according to primogeniture, where the oldest offspring is the first in the line of succession. This type of social inheritance of wealth is common in humans, but is less commonly found in animals (but see [70]). To implement this, I added new individuals directly below the youngest of their older siblings, or below their mother if they had no older siblings. In the second form of social inheritance, individuals inherit positions according to a general correlation between parent and offspring social position, reflecting genetic inheritance, parental effects or other processes that might lead parental and offspring hierarchy position to be similar [13,71,72]. To implement this condition, I randomly assigned offspring hierarchy position as either directly above or directly below the mother. Thus, like maternal rank inheritance with youngest ascendency, the magnitude of the difference between parent and offspring rank is always one position. This condition also represents a variant of maternal rank inheritance where daughters can acquire a position above their mothers, which occurs in some cases [51,73]. I once again fitted Markov chains to the hierarchy dynamics observed in these simulated societies. In the light of the results of the prior analysis of queenship effects, I treated the highest position in the hierarchy as its own state for these models. Like in previous analyses, I generated 10 000 hierarchy position trajectories for individuals starting in each state and summarized the mean and standard deviation of expected hierarchy position over time.

To assess the consequences of variation in social inheritance, I compared expected hierarchy position trajectories and inequality in expected reproductive output among societies with different types of inheritance. To calculate inequality in expected reproductive output, I used the same empirically informed reproductive probability function implemented in the simulations (see prior section and the electronic supplementary material) to calculate an expected reproductive output for each update of the simulation based upon the individual's hierarchy position during that update. Summing expected reproductive output across an individual's lifetime hierarchy position trajectory yielded the expected lifetime reproductive success for individuals starting in each state. I then used the Gini index, a widely used measure of inequality [74], to calculate inequality in expected lifetime reproductive success among individuals with different starting positions [75]. The Gini index ranges from 0 to 1, where 1 corresponds to maximal inequality. I repeated this process for each of the 10 000 trajectories generated in the prior analysis, producing a distribution of Gini indices for each type of simulated society. I then compared differences in the means of the distributions of Gini indices among societies with different types of inheritance.

### (e) Statistical analysis

Null hypothesis significance testing and use of *p*-values are not appropriate statistical approaches for simulation studies because sample sizes are arbitrary and the null hypothesis of no difference between groups is designed to be false by the programmer [76]. Instead, inference about differences between conditions are made by comparing effect sizes of different treatments, by comparing central tendencies of simulated data, or by visually inspecting model output. To compare predicted lifetime hierarchy trajectories from different Markov chains, I visually inspected trajectory plots and steady-state probabilities, and compared the median long-run hierarchy positions predicted by different steady state distributions.

## 3. Results

**Figure 1.**
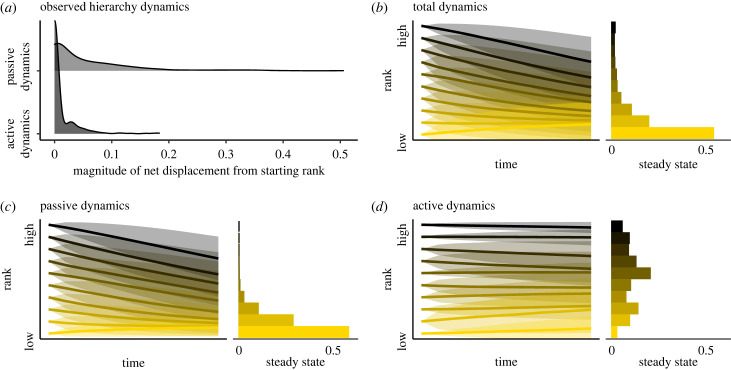
Hierarchy dynamics experienced by spotted hyenas. (*a*) Density plots of cumulative net displacement in hierarchy position owing to two different types of dynamics. Data are from 350 hyenas residing in four social groups. (*b–d*) Lifetime dominance trajectories for hyenas starting at each decile in the hierarchy, as predicted by the Markov chain of (*b*) all dynamics, (*c*) only passive dynamics, or (*d*) only active dynamics. In the left plots of (*b–d*), bold lines indicate mean predicted lifetime dominance trajectory and shading indicates one standard deviation range around the mean. Right plots show the steady state probability of being in each possible hierarchy decile in the long run, independent of starting state. Steady states are a mathematical property of the Markov process and can be interpreted as the states towards which individuals are drawn over time. (Online version in colour.)

Passive dynamics owing to demographic processes accounted for the majority of the dynamics observed in the longitudinal hierarchies of four groups of hyenas. In a sample of 350 individuals with dominance trajectories across their full tenure in the group (median = 2 years, range = 1–22 years), passive dynamics produced a mean cumulative change in hierarchy position equal to 5.7% of the hierarchy (i.e. net standardized rank change = 0.057), whereas active dynamics led individuals to change hierarchy positions amounting to a mean of only 1.0% of the hierarchy (net standardized rank change = 0.010; figure 1*a*; paired *t*-test *t*_349_= 11.28, *p* < 0.0001). The Markov chain analysis revealed a tendency for individuals to decline in rank over time, reflected in both the predicted lifetime dominance trajectories and the long-run steady states that describe the equilibrium point of the Markov process (figure 1*b*; median long-run hierarchy position decile = 1st, corresponding to the lowest 10% of the hierarchy). Fitting Markov chains to subsets of the empirical data revealed that passive dynamics are the primary driver of this pattern: hierarchy trajectories owing to passive dynamics showed the same pattern of decline (figure 1*c*; median long-run hierarchy position decile = 1st), whereas hierarchy trajectories owing to active dynamics did not (figure 1*d*; median long-run hierarchy position decile = 6th).

**Figure 2.**
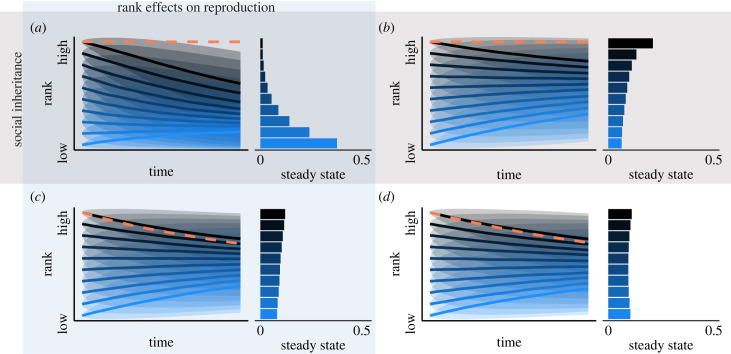
Hierarchy dynamics from simulated societies with (*a*) social inheritance of hierarchy position and rank effects on reproduction, (*b*) social inheritance of hierarchy position and random reproduction, (*c*) random placement of new individuals (no inheritance) and rank effects on reproduction, and (*d*) random placement of new individuals and random reproduction. In the left plots, bold lines indicate mean predicted lifetime dominance trajectory and shading indicates one standard deviation range around the mean. Dashed lines denote the mean trajectories of queens from a separate set of Markov chains that included queen (highest ranked individual) as a separate state. Right plots show the steady state probability of being in each possible hierarchy decile in the long run, independent of starting state. Steady states are a mathematical property of the Markov process and can be interpreted as the states towards which individuals are drawn over time. (Online version in colour.)

Results from simulated societies clarify how social inheritance shapes the passive dynamics produced by demographic processes. Simulated societies with hierarchical influence on reproduction and social inheritance showed the same pattern of hierarchy position decline observed in the empirical hyena data, leading to a long-run median hierarchy position in the 2nd decile of the hierarchy (figure 2*a*). In the absence of social inheritance of hierarchy position, individuals tended to regress towards mean hierarchy position, producing long-run steady states where the probability of occupying any rank state is roughly equal (figure 2*c,d*). Notably, when social inheritance of rank operates in the absence of rank effects, this pattern of regression to the mean is present but attenuated among the highest ranked individuals (figure 2*b*), leading to a slightly higher (7th) median hierarchy position decile than when there is no inheritance (with rank effect = 6th decile, figure 2*c*; no rank effect = 6th decile, figure 2*d*).

Re-fitting these Markov chains with a separate queen state revealed that in simulated societies with maternal rank inheritance, the queen is the only individual whose position in the hierarchy does not follow the pattern of the others (dashed lines in figure 2*a,b*). In these societies, the queen never changes position in the hierarchy, because it is impossible to inherit status above her. Regardless of the presence of a rank effect on reproduction, queenship is an absorbing state, meaning that once an individual transitions into the state of queen, they never subsequently transition to any other state. In societies without maternal rank inheritance, queens follow the same patterns as the others.

**Figure 3.**
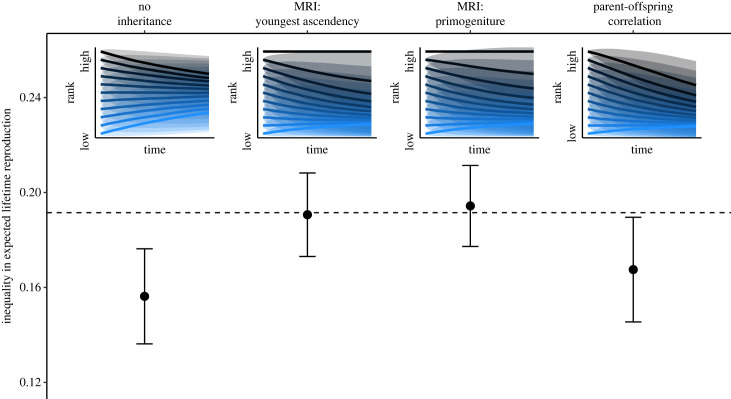
Hierarchy dynamics and reproductive inequality in simulated societies with different types of social inheritance (MRI, maternal rank inheritance). Insets: predicted lifetime dominance trajectories according to inheritance type. The highest rank state corresponds to the queens, and all others correspond to hierarchy deciles. Bold lines indicate mean predicted lifetime dominance trajectory and shading indicates one standard deviation range around the mean. Main plot: Gini indices quantifying reproductive inequality in societies with different inheritance types (mean and standard deviations). Dashed line corresponds to the inequality in reproduction implemented at each step in the simulation.(Online version in colour.)

Finally, explorations of societies with different variants of social inheritance of hierarchy position revealed that although all societies with rank effects on reproduction and social inheritance showed the previously documented pattern of decline in rank over time, the dynamics varied based on the specific details of social inheritance (figure 3, top). In societies with maternal rank inheritance, youngest ascendancy (younger siblings inherit above older siblings) produced a more rapid decline than primogeniture (older siblings inherit above younger siblings). Societies with parent-offspring correlation in hierarchy position did not show the queen effect, such that all individuals showed the same pattern in decline in rank over the life-course. Note that queens are treated as a seperate state for this analysis and are depicted by the highest line in figure 3.

These different dynamics manifested in variability in the inequality in expected lifetime reproductive success according to starting hierarchy position (figure 3, bottom). Both types of maternal rank inheritance produced greater reproductive inequality than societies without any social inheritance (youngest ascendency mean Gini = 0.19; primogeniture mean Gini = 0.19; no inheritance mean Gini = 0.16). Parent-offspring correlation in rank produced societies with reproductive inequality that was closer to societies with no inheritance (mean Gini = 0.17). Notably, hierarchy dynamics led to altered inequality in expected reproduction compared to the underlying inequality-generating process—in the simulations, inequality at each individual reproductive step had a Gini of 0.19 (figure 3*b*, dashed line). Dynamics reduced inequality in expected lifetime reproduction in societies with parent–offspring correlations in hierarchy position and those without social inheritance of hierarchy position, but not in societies with maternal rank inheritance.

## 4. Discussion

Here I demonstrate that the coupling of social inheritance of hierarchy position and rank effects on reproductive success produce structural effects on individual outcomes. Passive dynamics are the drivers of these effects, and in the case of hyenas, are also the largest contributor to changes in rank at the individual level (figure 1). Simulations reveal that, with the exception of the highest ranked member of the group, individuals decline in hierarchy position over their lifespans as a result of passive dynamics produced by demographic processes (figure 2). Removal of social inheritance of status or of rank effects on fitness eliminates this downward pattern, instead producing passive dynamics that drive a regression towards mean hierarchy position. Different types of social inheritance produced variation on the theme of downward decline, manifesting in differences in reproductive inequality (figure 3). In societies without inheritance or with a general parent-offspring correlation in status, passive dynamics produce lifetime reproductive inequality that is reduced compared to short-term reproductive inequality, because hierarchy dynamics lead to mixing of status over time (figure 3, dashed line, [77]). Maternal rank inheritance disrupts this process by differentiating the highest ranked individual from the rest of the group. Although the differences in reproductive inequality identified here are modest in magnitude, they are expected to accumulate across generations as offspring inherit starting ranks that are influenced by the dynamics identified in this study. In spotted hyenas, this manifests in dramatic differences in the persistence and average hierarchy position of different lineages over extended time-frames [41,78].

The results of this study contribute to understanding of how individual-to-society feedback loops operate within societies structured by social inheritance [1]. Prior work in these systems shed light on how inheritance of hierarchy position may have evolved [79,80] is achieved behaviourally [23,26,81], influences conflict and cooperation within these societies [39,82,83], and shapes social structure [84,85], but less is known about how this structure feeds back to influence individuals via society-to-individual effects. Here I identify structural consequences of living in a society with or without social inheritance of hierarchy position. Importantly, these are not the direct result of inheritance at the level of mother and offspring, but rather a result of living in a society where social inheritance is the norm. For instance, consider an individual who does not engage in social inheritance of status, and instead is added to the hierarchy at random—their lifetime position in the hierarchy will nevertheless be strongly shaped by the inheritance of status exhibited by other members of the group.

This work enhances our understanding of how demographic processes shape animal societies [2]. To build upon this work, it would be productive to explore how changes in group size and reproductive rate can modulate the effects observed here. When reproduction is slower and groups are larger, passive dynamics are expected to lead to more gradual change, because reproductive events are less common and a change of one position amounts to a smaller proportion of the hierarchy. In addition, previous simulation studies have suggested that shrinking groups lead to reduced adherence to Kawamura's rules of maternal rank inheritance with youngest ascendency [73], which would in turn alter the structural effects of social inheritance of hierarchy position (figure 3). The work presented here focused primarily on the reproductive side of demographic turnover, but future work could productively explore how variation in the distribution of mortality can also have structural effects [35,86–88]. However, work in hyenas and other species with nepotistic societies show either rank-independent mortality or a tendency for greater longevity among individuals at the top of the hierarchy [89–93]. This suggests that the assumption of rank-independent mortality is appropriate in some cases, and in others, incorporating rank-influences on mortality would further contribute to the pattern of rank-decline identified here by removing low-ranking individuals more frequently than high-ranking individuals.

These results reinforce the need for continued study of the dynamics of social hierarchies in order to gain a more complete picture of their evolution. A recent study of hierarchy dynamics in Tibetan macaques (*Macaca thibetana*) found an opposite pattern to what I observed here (although active and passive dynamics were not distinguished), where individuals tended to rise in rank over their life [66]. In a study of savannah baboons (*Papio cyanocephalus*), females showed a decline in rank as they age, but owing to active dynamics rather than passive dynamics as identified here [94]. Distinguishing between active and passive dynamics is important for facilitating understanding of how these different societies have evolved, because the evolution of competition over status is shaped by passive dynamics. For instance, in queuing systems, the tendency to rise in rank via passive dynamics dampens selection for risky status seeking behaviour [88], and in cooperative breeders, waiting for dominant breeders to die is a successful strategy by which subordinates ascend to become dominants [95]. The strong downward effects of passive dynamics identified here suggests that in hyenas, the benefits of active dynamics via competition over status are likely to be lost over time as a result of demographic turnover. One exception is if individuals can achieve the top-ranked position through status seeking, as queens are the only individuals whose hierarchy position is not affected by passive dynamics. This suggests that queenship, and competition over queenship, may be qualitatively different from other positions in the top of the hierarchy (e.g. [96]).

The Markov chain analytical approach implemented here and in [66] is useful for exploring these patterns, because it does not require data over the complete lifespan to make predictions about lifetime hierarchy trajectories or to identify long-run patterns in hierarchy dynamics. State transition probabilities are estimated solely based on changes in hierarchy position from one time period to the next, but the transition matrices can be used to generate expected transition state sequences of arbitrary length. Although this is a strength of the simple Markov chain model, implementing more complex Markov models offers a promising avenue for further insight. For instance, extensions of the Markov chain have been used to model time-dependent dynamics, where the probability of transitioning to a new state is influenced by time spent in the current state [65]. Higher-order Markov models can be used to assess how historical states influence state transitions, and hidden Markov models can be used to introduce covariates into estimates of state transition probabilities [67,97] or can be used to estimate transitions among latent states [98]. Consequently, future development and application of Markov models promises to be a productive avenue for better understanding social hierarchies.

Finally, this work contributes to an emerging perspective on social ageing in non-human animals, or changes in social connectivity over the life-course [99–101]. In some species with nepotistic societies, higher-ranked individuals are more socially well-connected than lower ranked individuals [85,102]. An insight from this work is that declines in social connectedness with age can result from society-to-individual effects of demographic events on hierarchy position.

## Data Availability

The data and code for reproducing this analysis are available from the GitHub Digital Repository: https://github.com/straussed/inheritance and from Zenodo digital repository: doi:10.5281/zenodo.7706570. The data are provided in the electronic supplementary material [103].
